# Genomic comparative analysis of the environmental *Enterococcus mundtii* against enterococcal representative species

**DOI:** 10.1186/1471-2164-15-489

**Published:** 2014-06-18

**Authors:** Guillermo D Repizo, Martín Espariz, Víctor S Blancato, Cristian A Suárez, Luis Esteban, Christian Magni

**Affiliations:** Instituto de Biología Molecular y Celular de Rosario (IBR-CONICET) and Departamento de Microbiología, Facultad de Ciencias Bioquímicas y Farmacéuticas, Universidad Nacional de Rosario, Suipacha 531, Rosario, S2002LRK Argentina; Departamento de Fisiología, Facultad de Ciencias Médicas, Universidad Nacional de Rosario, Santa Fe 3100, Rosario, S2002LRK Argentina

**Keywords:** *Enterococcus*, *Enterococcus mundtii*, Genomic comparative analysis

## Abstract

**Background:**

*Enterococcus mundtii* is a yellow-pigmented microorganism rarely found in human infections. The draft genome sequence of *E. mundtii* was recently announced. Its genome encodes at least 2,589 genes and 57 RNAs, and 4 putative genomic islands have been detected. The objective of this study was to compare the genetic content of *E. mundtii* with respect to other enterococcal species and, more specifically, to identify genes coding for putative virulence traits present in enterococcal opportunistic pathogens.

**Results:**

An in-depth mining of the annotated genome was performed in order to uncover the unique properties of this microorganism, which allowed us to detect a gene encoding the antimicrobial peptide mundticin among other relevant features. Moreover, in this study a comparative genomic analysis against commensal and pathogenic enterococcal species, for which genomic sequences have been released, was conducted for the first time. Furthermore, our study reveals significant similarities in gene content between this environmental isolate and the selected enterococci strains (sharing an “enterococcal gene core” of 805 CDS), which contributes to understand the persistence of this genus in different niches and also improves our knowledge about the genetics of this diverse group of microorganisms that includes environmental, commensal and opportunistic pathogens.

**Conclusion:**

Although *E. mundtii* CRL1656 is phylogenetically closer to *E. faecium*, frequently responsible of nosocomial infections, this strain does not encode the most relevant relevant virulence factors found in the enterococcal clinical isolates and bioinformatic predictions indicate that it possesses the lowest number of putative pathogenic genes among the most representative enterococcal species. Accordingly, infection assays using the *Galleria mellonella* model confirmed its low virulence.

**Electronic supplementary material:**

The online version of this article (doi:10.1186/1471-2164-15-489) contains supplementary material, which is available to authorized users.

## Background

The genus *Enterococcus* is a diverse group of low GC% gram-positive bacteria that contains over 33 species. The genera include commensal species of the gastrointestinal tracts of humans and animals, and also environmental strains that can be isolated from soil, surface waters and plant material [1, 2]. Enterococci are nutritionally fastidious microorganisms, which are associated with a large variety of human activities. In this sense, several strains have technological relevance since they are present in dairy, meat and other fermented foods, and some of them show probiotic effects [1, 2]. Nevertheless, they are not “generally recognized as safe” (GRAS) microorganisms for human consumption [U.S. Food Drug Administration (FDA) or European Food Safety Authority (EFSA)], even though they are phylogenetically related to the group of lactic acid bacteria (LAB). Particularly during the last decade, *Enterococcus faecalis* and *Enterococcus faecium* strains emerged as opportunistic human pathogens frequently associated with nosocomial infections with a high capacity to disseminate antibiotic resistance [3]. As a consequence, information on genetics and physiology of these species has increased dramatically in recent years; however, little data is available regarding other enterococci.

*Enterococcus mundtii* was described as a non-motile, yellow-pigmented enterococcus typically isolated from plant material, soil, cow teats and milker’s hands [4, 5] and infrequently associated to human infection [6]. As the rest of enterococci, they are facultative anaerobes and display a homolactic glucose metabolism. DNA GC content ranges from 38 to 39%, as determined by the melting temperature method [4]. We have recently announced the draft genome sequence of *E. mundtii* strain CRL1656 [7]. This strain was isolated from stripping milk of an Argentinean cow and, given its bacteriocinogenic capacity, it has been proposed as a probiotic microorganism to prevent mastitis in these mammals [8].

During the last decade, the availability of bioinformatics approaches for comparison of multiple bacterial genomes allowed the analysis of a huge amount of sequence data. In this sense, the aim of the present study was to gain insight into the similarities between the genetic content of *E. mundtii* and other enterococcal species and, more specifically, to investigate genes coding for virulence factors shared with *E. faecalis* and *E faecium* species, frequently behaving as opportunistic pathogens.

## Methods

### General inspection of the *E. mundtii*genome

Gene products putatively encoded by *E. mundtii* CRL1656 were identified using RAST [9] (GenBank accesion number: AFWZ00000000.1). In order to improve this sequence, a BLASTN approach (all versus all) was performed and those contigs shorter than 1,000 bp and with an homology higher than 99% with sequences already contained in a longer contig were deleted. In this manner, 87 contigs were removed from the database (approximately 200 Kb in total), resulting in a genome of 2.87 Mb (GC content 38.4%). The remaining contigs were ordered and oriented with Advanced Pipmaker using *E. faecalis* V583 genome as a reference [10]. Finally, contigs were concatenated, using a Perl script designed *ad hoc*, by including the sequence NNNNNCACACACTTAATTAATTAAGTGTGTGNNNNN, which harbors stop codons in all six reading frames [11].

After annotation of each of the draft genomic sequences in RAST (Table 1), as explained for *E. mundtii*, comparative genome analysis against other enterococcal species was performed. *E. mundtii* unique genes were determined by using RAST Compare Metabolic Reconstruction Tool [9].

**Table 1:** **Enterococcal species analyzed in this study and relevant features of their genomic sequences**

Organism	BioProject	Status	Size (Mbp)	GC%	Genes	Proteins (PubMed)	Proteins (RAST)	Putative pathogenicity determinants ^a^	Source	References
*E. mundtii* CRL1656	PRJNA71221	Scaffolds or contigs	2.87	38.4	2,646	2,589	2,589	659	Cow udder	[7]
*E. faecalis* V583	PRJNA57669, PRJNA70	Complete	3.36	37.4	3,412	3,264	3,363	1,006	Clinical	[10]
*E. faecalis* 62	PRJNA159663, PRJNA61185	Complete	3.13	37.4	3,157	3,075	3,075	876	Commensal	[12]
*E. faecium* XT16 (DO)	PRJNA55353, PRJNA30627	Complete	3.05	37.9	3,209	3,114	2,779	728	Clinical	[13]
*E. faecium* Com15	PRJNA55725, PRJNA32967	Scaffolds or contigs	2.77	38.2	2,783	2,724	2,773	755	Commensal	[14]
*E. italicus* DSM 15952	PRJNA61487, PRJNA53039	Scaffolds or contigs	2.31	39.2	2,455	2,405	2,275	700	Cheese	[15]
*E. casseliflavus* ATCC 12755	PRJNA63559, PRJNA53041	Scaffolds or contigs	3.55	42.4	3,606	3,548	3,415	942	Oral commensal	[14]
*E. gallinarum* EG2	PRJNA55685, PRJNA32927	Scaffolds or contigs	3.13	40.6	3,079	3,041	3,072	862	Clinical	[16]
*E. saccharolyticus* ATCC 43076	PRJNA206365, PRJNA191890	Scaffolds or contigs	2.60	36.9	2,625	2,582	2,594	780	Straw bedding	[15]

^a^According to predictions made using the Virulence Factors Data Base (see Methods).

### ANI versus shared genes with similar function plot

ANI values were calculated based on pairwise alignment of genome stretches using the JSpecies software with BLAST algorithm [17]. Calculation of ANI values is implemented as described by Goris J, *et al*. [18]. Shared genes with similar function among each enterococcal species described in Table 1 and *E. mundtii* were assessed using the Compare Metabolic Reconstruction tool from RAST server. Genes which were associated within a RAST subsystem for both microorganisms were considered as shared between them [9].

### Study of genomic islands

The analysis considered to detect putative Genome Islands (GEIs) was as follows. First, the regions detected by Alien-Hunter [19] were selected and secondly, positives results obtained either by Colombo/SIGI-HMM [20] or IslandPath-DIMOB [21] were taken as requirement for the next step. Regions identified using this approach were manually inspected using Artemis and DNAplotter [22] in order to find out further evidences related to GEIs, in general, and Pathogenic Island (PAIs), in particular [23]. A deviation in the G + C content frequency (calculated by DNAPlotter) plus the presence of insertion sequences and tRNA flanking regions jointly with transposases coding genes, which are important for DNA incorporation processes, were accepted as further evidence of the presence of a GEI. Lastly, specific features of each gene located within the putative PAI were determined. Particularly, genes involved in virulence, antibiotic resistance, and pathogenic mechanisms were traced, using the BLASTP tool in the Virulence Factors Data Base server [24]. Others functions of the features found in these regions were extracted from RAST annotations.

### Phylogenetic tree construction

This analysis involved sequences from nine enterococci species (Table 1) and the outgroup *Lactococcus lactis* SK11. Orthologous proteins were assigned using the OrthoMCL software [25]. In case that a particular enterococcal species contained more than one protein from the same group of orthologs, only the protein with the lower e-value was considered for the alignment. Concatenated orthologous protein sequences were aligned using ClustalX [26], and poorly aligned positions as well as excessively divergent regions were trimmed using GBlock 0.91b [27] resulting in a final alignment containing a total of 206,745 residues. Finally, the evolutionary history was inferred by using the Randomized Axelerated Maximum Likelihood algorithm (RAxML, [28]). DCMUT with empirical base frequencies and GAMMA distribution were used as substitution model. Reliability of the inferred tree was tested by bootstrapping with 1000 replicates.

### Analysis of regulators

The presence of orthologs for putative regulator proteins of *E. mundtii* CRL1656 in *E. faecalis* V583 and *E. faecium* DO genomes were determined as described in Additional file 1: Figure S1. Briefly, all protein sequences assigned as regulator proteins by RAST were used as query in a BLASTP search over *E. faecalis* V583 and *E. faecium* DO genome sequences. Cut-Off and Cut-In values were established as described in Additional file 1: Figure S1C. Then, *E. faecalis* V583 and *E. faecium* DO proteins sharing an identity percentage with *E. mundtii* CRL1656 proteins higher than the Cut-In value were considered as present in the *E. mundtii* genome while those with lower identity percentages were considered absent. Proteins with shared identity between Cut-In and Cut-Off values were further individually analyzed according to their gene context (Additional file 1: Figure S1D). For those *E. mundtii* CRL1656 proteins showing no homologs in *E. faecium* or *E. faecalis* a BLASTP was performed against a non-redundant database (RefSeq and Genbank) with a bit-score cut-off of 60.

### Semiqualitative comparison of potential virulence factors

For the enterococcal species described in Table 1, BLASTP analysis of their respective proteomes was made against the Virulence Factors Data Base [24]. The expectation value used as cut-off was 10^−10^. Only the first hit was recovered for each protein query.

### *Galleria mellonella*killing assay

Infection of *Galleria mellonella* larvae with *E. faecalis* was accomplished as previously described by Lebreton *et al*. [29]. Briefly, using a syringe pump (microliter #750, Hamilton), larvae (about 0.2 g and 3 cm in length) were infected subcutaneously with washed *E. faecalis* JH2-2 or *E. mundtii* CRL 1656 cells from an exponential culture in LBG administered in 5 μl of sterile saline buffer. Control groups of larvae received 5 μl of a saline solution only. In each test, 15 larvae were infected and the experiments were repeated at least three times. Larval killing was monitored up to 89 hours post-infection. Survival curves were constructed by the Kaplan-Meier method and compared by Log-rank analysis (R statistical software). P values of <0.05 were considered statistically significant [29].

## Results

### General features of the *E. mundtii*genome and phylogenetic analysis

**Figure 1:**
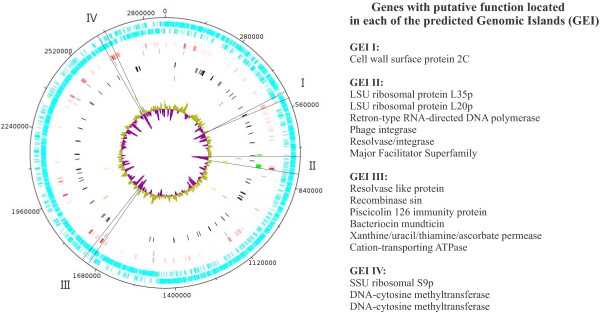
**Circular representation of the** ***E. mundtii*** **genome.** Starting from the outer circle: Rings 1 and 2 (ligth-blue) indicate positions of protein-coding genes on positive and negative strands, respectively. Ring 3 (red) shows presumably foreign DNA as predicted by Alien Hunter. Ring 4 (grey) shows alien genes predicted by Columbo Sigi. Ring 5 (green) corresponds to IslandPath-DIMOB predictions. Ring 6 (black) shows positions of tRNA genes. Ring 7 (blue), putative tranposases. Ring 8 (ochre), integrases. Ring 9 represents the G + C percentage, colored yellow for regions above median GC score (38%) and violet for regions less than or equal to the median. Circular sectors I-IV highlight the position of putative genomic islands. A list of genes with putative functions in each GEI is presented.

The genome sequence of *E. mundtii* strain CRL1656 was automatically annotated by using the RAST server [9]. A total of 2,589 coding sequences (CDS) and 57 structural RNAs (52 tRNAs) were predicted by this method. Putative biological roles have been assigned for 1,724 (67%) of the ORFs, whereas the remaining 865 (33%) encode hypothetical proteins for which no probable function could be predicted. The general features of the genome are summarized in Figure 1 and Table 1.

**Figure 2:**
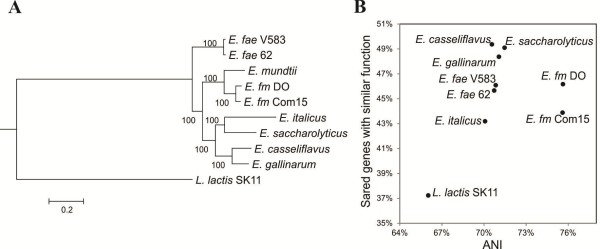
**Phylogenetic analysis of** ***E. mundtii*** **CRL1656. (A)** Core gene tree. ClustalX aligned sequences of core concatenated proteins were used for the phylogeny reconstruction analysis of enterococci species using the Randomized Axelerated Maximum Likelihood (RAxML) algorithm based on DCMUT with empirical base frequencies and GAMMA distribution model. The reliability of the inferred tree was tested by bootstrapping with 1000 replicates. The tree with the highest log likelihood (−2038246) is shown. Lactococcus lactis SK11 was included as outgroup species. **(B)** ANI plot. Shared genes with similar function and ANI values between E. mundtii and the bacterial genomes of the indicated strains are plot. *E. mundtii* CRL1656 (*E. mundtii*), *L. lactis* subsp. cremoris SK11 (*L. lactis*), *E. italicus* DSM15952 (*E. italicus*), *E. casseliflavus* ATCC12755 (*E. casseliflavus*), *E. faecalis* 62 (*E. fae* 62), *E. faecalis* V583 (*E. fae* V583), *E. saccharolyticus* ATCC 43076 (*E. saccharolyticus*), *E. gallinarum* EG2 (*E. gallinarum*), *E. faecium* Com15 (*E. fm* Com15), and *E. faecium* DO (*E. fm* DO). The data set supporting the results of this article is available in the TreeBASE repository, [http://purl.org/phylo/treebase/phylows/study/TB2:S15854].

*E. mundtii* CRL1656 phylogeny was analyzed with a tree generated from the concatenated sequences of 805 core proteins from nine enterococci and the outgroup *L. lactis* SK11 (Figure 2A). In this analysis we included representative enterococcal species of diverse origins, whose genomic sequences have been released (Table 1). We observed that *E. mundtii* is more related to *E. faecium,* which is frequently associated with nosocomial infections.

To further analyze the relationships among these enterococcal species, we constructed a plot of the shared genes with similar function between *E. mundtii* and the species under comparison versus average nucleotide identity (ANI) values (Figure 2B). ANI values are frequently used to verify prokaryotic species definitions [18, 30]. As expected, these values were lower than the 94% accepted as a threshold for species designation [18, 30]. *E. faecium* strains showed the highest ANI values (around 76%), with a content of shared genes with similar function between 44 and 46%. With respect to other enterococcal species, ANI varies within a narrow range (approximately 71%) and *E. mundtii* shared more genes with similar function with strains of non-clinical origin as *E. casseliflavus* ATCC12755 and *E. saccharolyticus* ATCC 43076 than the rest of the analyzed strains.

We were also interested in performing a bioinformatic quantification of genes horizontally transferred to the *E. mundtii* genome. According to the Alien-Hunter software, 39% of the genome was acquired by horizontal transfer. A list of genes coding for integrases, transposases and phage-related proteins found by the RAST annotation is given in Additional file 2: Table S1. In this regard, four gene clusters that fit the criteria as GEIs (refer to Methods for details) were detected and are pointed out in Figure 1. GEI I contains a putative cell wall surface anchor protein, that could be involved in adhesion and invasion mechanisms. Interestingly, a gene for the bacteriocin mundticin was detected in the GEI III cluster, which is genetically linked to its immunity protein and transporter.

Regarding genetically distinctive features of *E. mundtii*, a comparative analysis performed between strain CRL1656 and the other enterococcal species included in this study (Table 1) revealed a set of 22 unique proteins with putative functions for this microorganism (Table 2). Among this group of proteins, besides the mundticin previously mentioned, two proteins that could be responsible for capsule biosynthesis (see Virulence factors and antibiotic resistance section) and a toxin-antitoxin MazE-MazF system were detected.

**Table 2:** **Unique genes among enterococcal species found in the** ***E. mundtii*** **genome**

Feature ID	Start	Stop	Length (AA)	Putative function	GEI
fig|6666666.25220.peg.49	39017	39349	111	Putative EsaC protein analog (Listeria type 3)	
fig|6666666.25220.peg.389	768326	768559	78	Programmed cell death antitoxin MazE	
fig|6666666.25220.peg.401	780662	782068	469	Retron-type RNA-directed DNA polymerase (EC 2.7.7.49)	
fig|6666666.25220.peg.507	875260	875538	93	Zinc finger domain-containing protein	
fig|6666666.25220.peg.523	889680	889808	43	Probable GTPase related to EngC	
fig|6666666.25220.peg.751	1130121	1131056	312	Mtx2	
fig|6666666.25220.peg.781	1159802	1160566	255	Hnh endonuclease	
fig|6666666.25220.peg.928	1322901	1323290	130	Holin	
fig|6666666.25220.peg.1128	1488458	1487922	179	Rhs-family protein	
fig|6666666.25220.peg.1403	1721023	1720727	99	putative piscicolin 126 immunity protein	III
fig|6666666.25220.peg.1405	1723390	1723214	59	Bacteriocin mundticin	III
fig|6666666.25220.peg.1456	1777938	1778441	168	Phosphoribosylanthranilate isomerase like (EC 5.3.1.24)	
fig|6666666.25220.peg.1618	1965831	1965595	79	COG0477: Permeases of the major facilitator superfamily	
fig|6666666.25220.peg.1862	2217937	2219130	398	ATPase	
fig|6666666.25220.peg.1907	243734	245122	463	endo-beta-galactosidase, GlcNAc-alpha-1,4-Gal-releasing	
fig|6666666.25220.peg.1961	2301444	2302463	340	PE-PGRS family protein	
fig|6666666.25220.peg.2071	2406250	2405168	361	capsular polysaccharide biosynthesis protein Cps4G	
fig|6666666.25220.peg.2073	2408118	2407363	252	putative capsule biosynthesis protein	
fig|6666666.25220.peg.2156	2483290	2482904	129	Putative EsaC protein analog (Listeria type 3)	
fig|6666666.25220.peg.2203	2515077	2514832	82	SMT0609 replicon stabilization protein (antitoxin to SMT0608)	
fig|6666666.25220.peg.2352	2693714	2693941	76	COG0477: Permeases of the major facilitator superfamily	
fig|6666666.25220.peg.2403	2758856	2757387	490	Internalin-like/N-acetylmuramoyl-L-alanine amidase	

### Identification of a gene encoding a putative bacteriocin

A cluster of three genes involved in bacteriocin synthesis and transport, which is unique to this microorganism, was found. These genes were designated *munA*, *munB*, and *munC*. The structural gene *munA* encodes a 58-aminoacid mundticin precursor. The mature peptide is a class IIa mundticin with an estimated molecular weight of 4,289 Da and pI of 9.45. The BLASTP protein database homology search on the 43-amino acid deduced mature peptide, mundticin 1656, revealed that its sequence is identical to that of the plasmid encoded-mundticin KS from *E. mundtii* NFRI7393 [31] and a 95% identical to that of *E. mundtii* ATO6 [32]. The pre-peptide contains a leader peptide of 15 amino acids with a consensus GGXaa processing site. The mature peptide contains the YGNGV motif at positions 3–7, characteristic of class IIa bacteriocins. Moreover, it contains the two cysteine residues (C-9 and C-14) forming the disulfide bridge, which are well conserved in all class IIa bacteriocins [33].

The *munB* gene encodes a 674-amino acid polypeptide, which exhibits 98% of similarity with the 674-amino acid ABC transporter MunB found in *E. mundtii* NFRI7393, involved in the maturation and excretion of enterocin KS [31]. The *munC* gene encodes a mundticin immunity protein of 98 amino acids, as evidenced by the close similarity to the immunity protein of mundticin KS [31].

### Stress response systems

One of the most relevant characteristics of the *Enterococcus* genus is its capacity to resist and grow at low pH. Thus, we analyzed the presence of genes involved in the acidic stress response in the *E. mundtii* genome. We identified eight genes encoding the different subunits of the F_0_F_1_-ATPase, which is also present in the enterococcal core genome previously described. This complex is involved in pH homeostasis through the generation of proton motive force by ATP consumption [34]. The organization of these genes was the same as that for other enterococcal F_0_F_1_-ATPases (*atpBEFHAGDC*), where *atpBEF* encode the A, C and B subunits of the membrane-bound F_0_ domain, whereas *atpHAGDC* genes encode the δ, α, γ, β y ϵ subunits of the cytoplasmic F_1_ domain, respectively [34]. In addition, the genome of *E. mundtii* possesses an operon constituted by nine genes (F, I, K, E, C, G, A, B and D) homologous to the *ntp* genes of *E. hirae* [35] encoding a Na^+^-pumping V_1_V_0_-ATPase. The V_1_ domain is a peripheral complex responsible for ATP hydrolysis, and is constituted by A, B, C, D, E, F and G subunits. The V_0_ domain is an integral complex responsible for Na^+^ translocation across the membrane.

Furthermore, three gene clusters also indirectly involved in resistance to low pH were identified. One of them corresponds to the *cit* genes, homologous to those recently characterized in *E faecalis*, which are responsible for the degradation of citrate and are organized in two divergent putative operons [36–39]. The first transcriptional unit contains genes encoding a GntR-family transcriptional regulator and a citrate permease belonging to the Me^2+^-CitMHS family (*citO* and *citH* genes, respectively). In the second operon, genes responsible for the codification of citrate lyase subunits (*citDEF*), citrate lyase accessory genes (*citC* and *citX*) and a membrane oxaloacetate decarboxylase (*oadAHBD*) were found. This genetic organization resembles that found in *E. faecium*, which differs from the *E. faecalis cit* locus in the position of another citrate lyase-accesory gene, *citG*, and also in the absence of the soluble oxaloacetate decarboxylase gene, *citM*. The second group of genes involves the *mleS* (malolactic enzyme), *mleT* (malate transporter) and *mleR* (transcriptional activator from LysR family) genes, which are required for the malolactic fermentation and show homology to those found in *E. faecium* [40]. Finally, genes associated to the arginine deiminase pathway (ADI), which generates ATP and contributes to low-pH resistance, were identified. These genes are present in the core genome of selected enterococci and code for the three main enzymes of the system, arginine deiminase, ornithine transcarbamylase and carbamate kinase. Additionally, we identified a homologue to *arcR* divergently oriented, which encodes an activator of the ADI system (Crp-Fnr family of regulators). Besides, a homologue of the arginine-ornithine transporter (*arcD*) of *Lactococcus garvieae* ATCC 49156 was identified in another genomic region of *E. mundtii*.

Other related proteins required for optimal stress resistance were identified in the genome of *E muntdii*: RecA, a mediator of homologous recombination and regulator of the SOS response, chaperonins GroEL and GroES, HtrA, a protein involved in proteolysis of abnormal proteins synthesized under stressful conditions, the enzymes involved in the synthesis of D-alanyl-lipoteichoic acid (*dlt* operon, see Table 3), and the diacylglycerol kinase DagK, involved in acid resistance [34].

**Table 3:** **Putative virulence factors present in** ***E. mundtii***

Virulence factor	Genes	Homologous locus	Role in pathogenesis	Reference
Adhesin				
	*scm*	EfaeDRAFT_0418	Adherence	[41]
Pili				
	*ebpABC*	EF1091-EF1093	Biofilm formation	[42]
	*ebpR*	EF1090	*ebp* locus regulation	[43]
	*strC*	EF0194	Pilus Sortase C	[42]
	*strA*	EF3056	Pilus Sortase A	[44]
	*rnjB*	EF1185	*ebp* locus regulation	[45]
Capsule				
	*eps locus*	EFSG_00424, −25, −26, −31, −33, −35 to −41	Resistance to phagocytosis	[16]
Cell wall				
	*galU*	EF1746	Resistance to multiple types of stress	[46]
	*bgsAB*	EF2891- EF2890	Adherence and biofilm formation	[47]
	*dlt* locus	EF2749-EF2746	Biofilm formation	[46]
	*bopD*	EF0954 (MalR)	Biofilm formation	[48]
	*epa* locus	EFWG_01395-EFWG_01370	Biofilm and resistance to PMNs	[49]
	*sagA*	EfaeDRAFT_1606	Adherence	[50]
Others				
	*efafm*	EfaeDRAFT_2037	Adherence	[43]
	*msrA*	EF1681	Oxidative stress	[51]
	*msrB*	EF3164	Oxidative stress	[51]
	*gls33*	EfaeDRAFT_2384	Stress response protein	[52]
	*gls20*	EfaeDRAFT_2389	Stress response protein	[52]
	*sigV1* and *sigV2*	EF3180	Mouse bacteremia	[53]
	*eep*	EF2380	Biofilm formation	[54]

### Analysis of regulators

Since adaptation to different niches requires a fine-tuning of gene expression, we searched for regulators responsible for sensing the bacterial environmental milieu and physiological state. By knowing the set of regulators shared by related bacteria and detecting the presence of those that are unique, it is possible to infer the common and specific characteristics of niches and lifestyle among them. In the attempt to study the complete set of regulators encoded by the *E. mundtii* genome, a database consisting of 122 genes was constructed (see Methods section) and a comparative analysis against *E. faecalis* V583 and *E. faecium* DO was performed.

#### Core regulators

58 (48%) of the analyzed genes were found in the three genomes (Additional file 1: Figure S1). Although 24 of these genes are not assigned to any RAST subsystems, the rest of them were implied in diverse functions including sugar uptake and utilization (mannose, lactose, galactose, D-tagatose and galactitol), anabolic pathways (purine, pyrimidine, deoxyribonucleotides, glutamine, glutamate, aspartate and asparagine, fatty acids and pyridoxin), amine compounds degradation (arginine, ornithine, polyamine, chitin and N-acetylglucosamine), ATP-dependent proteolysis, phosphate uptake, competence, heme and hemin uptake and utilization systems, metal homeostasis/resistance (copper, cobalt, zinc and cadmium) and oxidative stress (Additional file 3: Table S2, Core regulators).

#### Non-core regulators

37 genes (30%) of the analyzed regulators were found in *E. mundtii* and *E. faecium* (20%; 25 genes) or *E. faecalis* (10%; 12 genes) but not in the three genomes at the same time (Additional file 1: Figure S1). Although higher cut-in and cut-off values were used for ortholog assignment to *E. faecium* with respect to *E. faecalis* (refer to Methods section)*,* higher numbers of orthologs were found in the former, which correlates with the closer phylogenetic relationship between the species. While 25 out of the 37 genes are not assigned to any RAST subsystems, the remaining 12 were assigned to diverse functions including sugar uptake and utilization (fructooligosaccharides, raffinose, lactose and galactose), heme and hemin uptake and utilization systems, degradation of amine compounds (arginine and ornithine), organic acid metabolism (pyruvate and sialic acid) and oxidative stress (Additional file 3: Table S2. Non-core regulators).

#### E. mundtii species-specific regulators

27 gene orthologs (22%) could not be found in *E. faecium* or *E. faecalis,* and 12 (10%) of these genes have orthologs in other LAB. The remaining genes only have orthologs in phylogenetically more distant bacteria (*Bacillus, Clostridium, Listeria*). Although none of these genes belong to a RAST subsystem, a preliminary sinteny analysis assigned them to functions related to carbon compound utilization (maltose, xylose, cellobiose and formaldehyde), anabolic pathways (sulfur containing compounds, coenzyme A, lysine), cell surface functions, ABC-type transporters and other roles (hydrolase, DNA methylation, ribosomal related) (Additional file 3: Table S2).

Among all the transcriptional regulators found in the *E. mundtii* genome, there is a subset corresponding to response regulators (RR) putatively forming part of two-component signal transduction systems (TCS). The importance of TCS for the ability of *E. faecalis* to respond to environmental stimuli has been previously stressed by Hancock and Perego [55]. The *E. mundtii* genome contains putative genes coding for 17 TCS and one orphan response regulator (Additional file 4: Table S3). We found *E. faecalis* homologues for 14 of the *E. mundtii* TCS and also for the orphan RR. As a general rule for most bacterial sensor kinases, all of the HK in *E. mundtii* were predicted to be membrane localized (data not shown). Among the remaining HK, one (IIb) showed homology to proteins present in environmental enterococci, and HKs IIIa-i and j presented homology to sensors encoded by *E. faecium*. This analysis suggests that the set of TCS present in *E. mundtii* is highly similar to that encoded by other enterococcal species.

### Identification of genes involved in pigmentation

*E. mundtti* CRL1656 shows a characteristic yellowish pigmentation on agar plates, originated by the production of carotenoids as reported for other *Enterococcus* species [56]. Genes responsible for carotenoid production have been previously described in *Staphylococcus aureus* [57] and *Lactobacillus plantarum* [58]. It has been shown that *crtM* and *crtN* constitute a bicistronic operon encoding the enzymes responsible for the production of the yellow pigment, presumably the C30 carotenoid 4,4′-diaponeurosporene. In *S. aureus*, diaponeurosporene is further converted to staphyloxanthin, the orange carotenoid present in most staphylococci strains. For this, *S. aureus* harbors up to three extra enzymes coded by genes *crtO*, *crtP* and *crtQ*, which are located in the same operon as *crtM* and *crtN* (*crtOPQMN)* [57]. The *E. mundtii* genome encodes a protein with 68 and 77% similarity to CrtN from *S. aureus* and *L. plantarum*, respectively. Regarding CrtM, the homology is reversed, with the homologue from *L. plantarum* sharing a similarity higher than the one corresponding to *S. aureus* (64 versus 53%, respectively). Remarkably, this analysis also detected the presence of putative homologues for *S. aureus* CrtO (60%), CrtP (73%) and CrtQ (52%). However, genes coding for these proteins are located in a different cluster with respect to *crtN* and *crtM*, and also show a different organization (*crtPQO*) to that found in *S. aureus* [57]. Furthermore, a comparative analysis with other enterococcal species indicated that *crtN* and *crtM* homologues were present in *E. gallinarum*, *E. saccharolyticus* and *E. casseliflavius* genomes. Noteworthy, putative proteins involved in staphyloxanthin biosynthesis were also detected in *E. casseliflavius*.

### Virulence factors and antibiotic resistance

The potential virulence genes encoded by the genome of *E. mundtii* are of particular interest*.* An initial comparative analysis between different enterococcal species using the Virulence Factors Data Base [24] revealed that *E. mundtii* has a reduced number of putative virulence determinants (Table 1). We searched for virulence genes previously analyzed by *G. mellonella* or mice infection models [3, 46, 59] to validate our results (Table 3). Among collagen adhesins, only the *E. faecium* widely spread *scm* gene [41] was detected in the *E. mundtii* genome, which lacks the most extensively studied *ace* and *acm* genes. We also found the *ebp* pili coding cluster, in conjunction with its cognate transcriptional regulator EbpR [43] along with sortase proteins [42, 44], which are ubiquitous in *E. faecalis* and *E. faecium*. Furthermore, we identified the *rnjB* gene, which encodes a putative RNase J2 that activates the transcriptional expression of the *ebpABC* operon [45]. Noteworthy, expression of the *ebp* locus is negatively regulated by the Fsr system [60], which is also present in *E. mundtii* (Additional file 4: Table S3). None of the other pili-encoding loci previously reported for *E. faecalis* (*bee* plasmidic genes, [61]) or *E. faecium* [41] were detected. A cluster of genes that might be responsible for the synthesis of capsular polysaccharides (*eps* locus, Additional file 5: Figure S2) was identified. It codes for proteins with homology to that reported in *E. faecium* [16]. In *E. mundtii*, we found three of the four phosphoregulatory system proteins conserved in all species of *E. faecium* (the LytR-Csp-Psr family protein is absent). Furthermore, two glycosyl transferases and the conserved dehydrogenase and flippase were detected as well as several conserved PTS-related proteins linked to the *eps* cluster in *E. faecium* 1,141,733 (Additional file 6: Table S4).

Moreover, several virulence genes that are related to cell wall synthesis in *E. faecalis* were found (Table 3). Remarkably, genes responsible for the synthesis of Epa (enterococcal polysaccharide antigen, suggested to be associated to the cell wall) were detected (Additional file 5: Figure S2). The core genes showed a similar genetic organization to that observed in *E. faecium* [16]. In *E. mundtii* the variable accessory region encode predicted glycosyltransferases and other proteins with potential roles in WTA production (encoded by *tag* genes), and the genetic organization resembles that of *E. faecium* Com15, a microorganism of human intestinal origin (Additional file 6: Table S4). Since major growth defects have been detected for mutants in different genes of this locus, Rigottier *et al.* [46] have proposed that these genes are important for the fitness of the bacterium rather than bona fide virulence factors.

Other virulence genes detected correspond to *msrA* and *msrB*, encoding methionine sulfoxide reductases important for the oxidative stress response, macrophage survival, and persistent infection [51]; and *gls* genes encoding general stress proteins (Gls33 and Gls20), important for adaptation to the intestinal environment and in mouse peritonitis models [52]. Moreover, this analysis detected the presence of a gene with significant homology to *bopD,* coding for the transcriptional regulator of maltose metabolism, which has been implicated in biofilm formation and bacteremia in mice [48].

Regarding resistance to antibiotics, the search for vancomycin resistance genes (*vanA, vanB, vanC, vanD* and *vanE*), tetracycline *(tetM, tetL, tetS, tetO, tetK*, and *tetW*) gentamicin (*aac* (6′)-*aph* (2′)), chloramphenicol (*cat*), lincosamide (*lnuA*), erythromycin (*ermT* and *ermB*), methicillin (*mecA*) and penicillin (*blaZ*) did not identify genes with significant homology (Additional file 7: Table S5). Furthermore, mutations conferring resistance to ampicillin or ciprofloxacin within the genes coding for *gyrA* or *pbp5,* respectively, were neither identified.

### *E. mundtii*effect on *G. mellonella*survival

In order to study *E. mundtii* virulence, CRL1656 strain was used to infect the insect host model *Galleria mellonella* [29, 46]. *G. mellonella*, is a reliable model host to study the pathogenesis of numerous human pathogens [62]. The capacity of a pathogen to kill *G. mellonella* has a correlation with virulence in mammalian models [62]. In fact, the innate immune systems of *Galleria* larvae and mammals share a high degree of homology [63].

**Figure 3:**
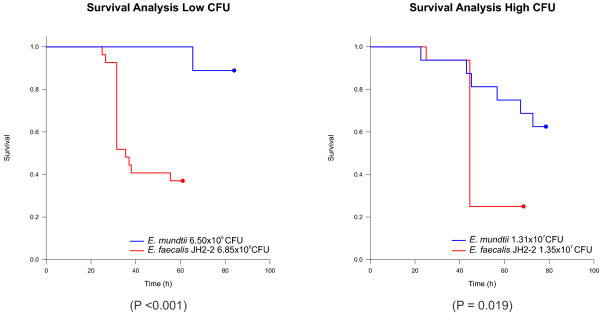
***E. mundtii*** **effect on** ***G. mellonella*** **survival.** Kaplan-Meier survival analysis of *G. mellonella* upon infection with *E. mundtii* (blue lines) and *E. faecalis* JH2-2 (red lines). Data are representative of three separate survival experiments performed with groups of 15 insects for each. Survival curves were constructed by the Kaplan-Meier method and compared by Log-rank analysis. P values of <0.05 were considered statistically significant.

In our analysis, the *E. faecalis* JH2-2 strain was used as control [64]. This strain was initially isolated from a nosocomial infection, and constitutes a genetic model extensively used in our laboratory [36, 38, 40, 65] and by others [66, 67]. The semiquantitative analysis performed (as described in the methods section) on the predicted proteins of the JH2-2 genome revealed 874 hits for putative virulence factors). As shown in Figure 3, *G. mellonella* survival was significantly LogRank test, P < 0.05 greater after infection with *E. mundtii* than with *E. faecalis* JH2-2 strain, at two different enterococcal concentrations. In the control groups infected with sterile saline buffered solution no larvae died in any of the replicates (data not shown). As consequence, these results confirm that *E. mundtii* is not as effective as *E. faecalis* in colonizing and killing *G. mellonella* larvae.

## Conclusion

In this report, a study of the genomic data of *E. mundtii* CRL1656 is presented for the first time. Additionally, a comparative analysis including the full genomic sequence of representative enterococcal species of diverse origins was conducted (Table 1). *E. mundtii* CRL1656 contains 805 CDS in common with other species of *Enterococcus* and only a low number of unique CDS, among which we identified a cluster encoding a bacteriocin and other related proteins. A phylogenetic tree for concatenated sequences of enterococcal core proteins was constructed (Figure 2A), indicating that *E. mundtii* is closer to *E. faecium*. This is in accordance with the *sodA* gene sequence phylogenetic comparison reported by Poyart *et al.* [68], which showed that these two enterococcal species cluster together. Moreover, as shown in Figure 2B, the highest ANI percentage was found between these microorganisms.

Bacterial genome plasticity is influenced by the presence of GEIs, which may include genes encoding for any number of functions, notably pathogenicity determinants [3, 46, 59]. Only 4 putative GEIs were identified in strain CRL1656, and none of them carries genes that might be encoding known virulence determinants. Many virulence genes and their functions have been described in enterococci [3, 46, 59]. Our bioinformatic analysis revealed the lowest number of putative pathogenicity factors for *E. mundtii* in comparison to other *Enterococcus* strains (Table 1), including different species from diverse origins (clinical, food and commensal). However the analysis of the presence or absence of virulence traits is not decisive to determine the commensal or pathogenic nature of a bacterium and the predictive methods have limitations and are not sufficient to conclude on the pathogenesis of the microorganism. This observation clearly arises from the analysis of Table 1, in which the commensal strain *E. faecium* Com15 possesses a higher number of putative virulence determinants (755) than the clinical isolate *E. faecium* strain DO (728). In this study, the virulence of the *E. mundtii* CRL1665 strain was evaluated using the *G. mellonela* model. As shown in the Figure 3, *E. mundtii* CRL1665 resulted non-virulent at low dose whereas the JH2-2 strain used as control killed 60% of larvae at 60 h. At high dose similar results were found *E. mundtii* strain was able to kill only 40% of larvae, whereas the control killed near the 80%. Our data indicate that the *E mundtii* strain CRL1665 as it was predicted by bioinformatic analysis is less virulent than the model *E. faecalis* JH2-2.

Remarkably, neither homologues for the main secreted factors shown to be critical for enterococcal pathogenesis, *sprE* (serine protease) and *gelE* (gelatinase), were found in *E. mundtii* nor other relevant genes such as *cyl* (transport and activation of a cytolisin [69]), *agg,* (adherence to eukaryotic cells) and *esp* (cell wall protein involved in immune evasion [70]). Interestingly, a gene annotated as a putative hemolysin was found. This protein contains a domain belonging to the hemolysin III family (Pfam 03006), which includes proteins from pathogenic and non-pathogenic bacteria, *Homo sapiens* and *Drosophila melanogaster*. In *Bacillus cereus*, it has been shown to function as a channel-forming cytolysin. In contrast, the cytolytic hemolysins commonly expressed by many enterococcal bacteraemia isolates, are two-component peptide systems (CylL_L_ and CylL_S_) whose expression requires the products of an eigth-gene locus [71]. However, this locus is absent in *E. mundtii*. Experimental assays will be needed to define if the type III-hemolysin has a lytic role *in vivo*.

Some enterococcal species, particularly those thought to be associated with soil and non-human hosts [72], including *E. mundtii,* contain yellow pigments*.* Pigments in *E. mundtii* were identified as carotenoids [73]. This study has shown the presence of genes involved in the C30 carotenoid biosynthetic pathway in this bacterium, which is also present in most of the environmental enterococcal sequenced species. The ubiquitous detection of *crtM* and *crtN* genes, involved in the biosynthesis of the yellow 4,4’-diaponeurosporene, as well as the documented carotenoid production by these microorganisms [4], suggest that the role of carotenoids in enterococcal environmental fitness must be important. Carotenoids function as protectors against photodamage as they are able to quench ROS. Pigmented enterococci are protected from solar inactivation and can persist for extended periods of time in marine water relative to non-pigmented species [56]. Therefore, we can speculate that pigmentation in *E. mundtii* could play a protective role when the microorganism is exposed to the environment.

In this work we also identified those genes encoding the machinery responsible for the production of a bacteriocin previously reported by Espeche *et al.* [8]. This feature constitutes a relevant and unique characteristic of the strain. With respect to its biological activity, mundticin 1656 was shown to inhibit the growth of *Listeria monocytogenes* and a variety of lactic acid bacteria [8]. Noteworthy, the use of bacteriocins for the prevention or treatment of mastitis in cows has the potential to reduce the dependence on antibiotics. Given the genetic features observed throughout this work, *E. mundtii* could be used to this end, but this hypothesis needs to be further investigated.

## Electronic supplementary material

Additional file 1: Figure S1: Search for orthologs of *E. mundtii* CRL1656 putative regulators in *E. faecalis* V583 and *E. faecium* DO genomes. **A)** General workflow. *E. mundtii* CRL1656 regulators were used as query in a BlastP search over *E. faecalis* V583 and *E. faecium* DO genomes. Obtained data was used to define Cut-In and Cut-Off values and subsequently to determine the presence or absence of each regulator in both genomes. **B)** Construction of a Correlation Table. Retrieved sequences from previous BlastP analysis with more than 80% coverage, score values higher than 50 and sharing the highest degree of identity were directly added to the Table. **C)** Workflow for Cut-In and Cut-Off definition. *E. faecalis* V583 or *E. faecium* DO proteins correlating with more than one *E. mundtii* CRL1656 protein were used as Input Data. Each set of *E. mundtii* CRL1656 proteins was sorted by percentage of identity (Id%). Those with the highest value and more than 50% of Id% were defined as orthologs and used for Cut-In definition. The remaining proteins were defined as paralogs and used to set up the Cut-Off value. Protein sets with the highest Id% but lower than 50% were analyzed individually to determine whether they were paralogs or orthologs. Finally, the lowest Id% value among all orthologs was defined as the Cut-In value and the highest Id% among all the paralogs was defined as Cut-Off value. **D)** Workflow for Ortholog Table construction. The pruned correlation table results from eliminating paralogs defined in **(C)** from the correlation table defined in **(B)**. All proteins that shared an Id% higher than the Cut-In value were considered as present and those with Id% lower than the Cut-Off were considered absent. Presence or absence of proteins with shared identity between Cut-Off and Cut-In values was analyzed individually by sinteny. (PDF 407 KB)

Additional file 2: Table S1: Putative functions associated to horizontal gene transfer. (PDF 405 KB)

Additional file 3: Table S2: Transcriptional regulators encoded in the *E. mundtii* genome. (XLS 34 KB)

Additional file 4: Table S3: Two-component systems present in *E. mundtii*. (XLS 270 KB)

Additional file 5: Figure S2: *epa* and *eps* gene clusters present in *E. mundtti* CRL1656. *E. faecium* 1.141.733 *epa* genes and *E. faecium* Com 15 *eps* genes are shown for comparison. Genes are colored following the code included in the figure. (DOC 59 KB)

Additional file 6: Table S4: *Eps* and *epa* loci in *E. mundtii*. (XLS 31 KB)

Additional file 7: Table S5: Antibiotic resistance. (XLS 28 KB)
