# Supplementary figures and images for: Genomic comparative analysis of the environmental Enterococcus mundtii against enterococcal representative species
Source: BMC Genomics. 2014 Jun 18;15(1):489. doi: 10.1186/1471-2164-15-489 (PMC4076982; doi:10.1186/1471-2164-15-489)

A)

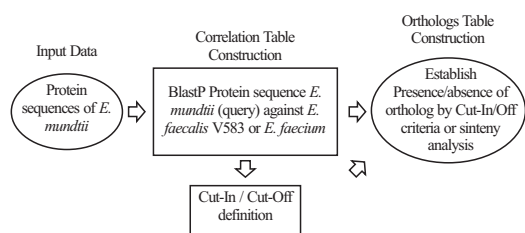

B)

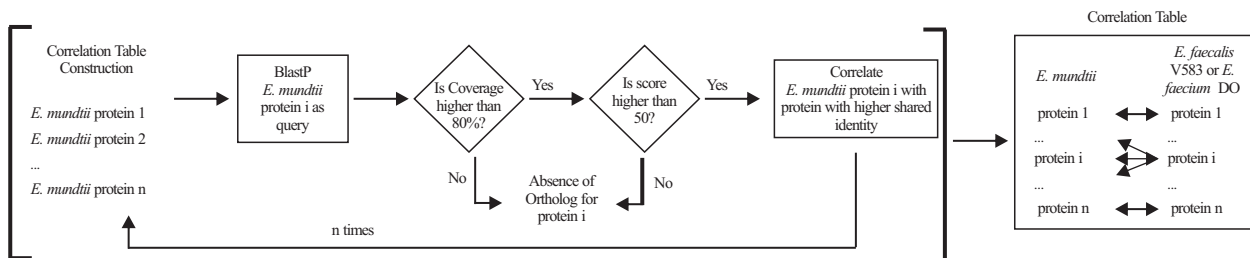

C)

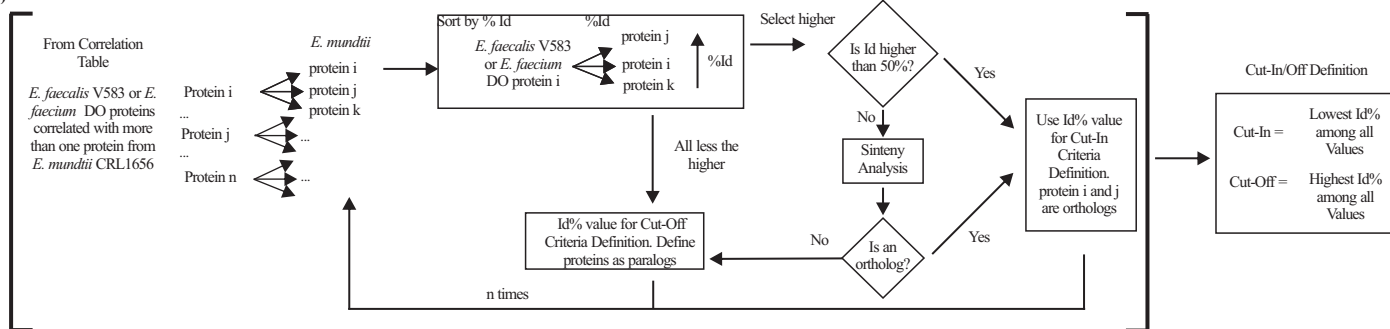

D)

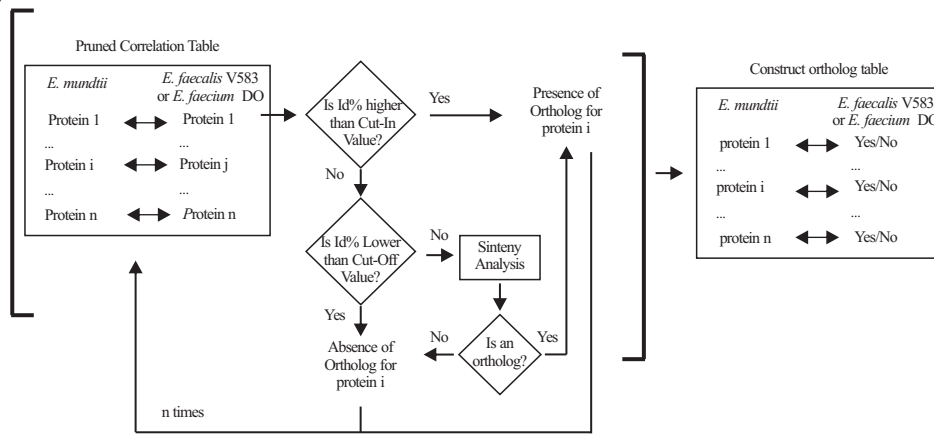

Supplement: Supplementary file 1 — Additional file 1: Figure S1: Search for orthologs of E. mundtii CRL1656 putative regulators in E. faecalis V583 and E. faecium DO genomes. A) General workflow. E. mundtii CRL1656 regulators were used as query in a BlastP search over E. faecalis V583 and E. faecium DO genomes. Obtained data was used to define Cut-In and Cut-Off values and subsequently to determine the presence or absence of each regulator in both genomes. B) Construction of a Correlation Table. Retrieved sequences from previous BlastP analysis with more than 80% coverage, score values higher than 50 and sharing the highest degree of identity were directly added to the Table. C) Workflow for Cut-In and Cut-Off definition. E. faecalis V583 or E. faecium DO proteins correlating with more than one E. mundtii CRL1656 protein were used as Input Data. Each set of E. mundtii CRL1656 proteins was sorted by percentage of identity (Id%). Those with the highest value and more than 50% of Id% were defined as orthologs and used for Cut-In definition. The remaining proteins were defined as paralogs and used to set up the Cut-Off value. Protein sets with the highest Id% but lower than 50% were analyzed individually to determine whether they were paralogs or orthologs. Finally, the lowest Id% value among all orthologs was defined as the Cut-In value and the highest Id% among all the paralogs was defined as Cut-Off value. D) Workflow for Ortholog Table construction. The pruned correlation table results from eliminating paralogs defined in (C) from the correlation table defined in (B). All proteins that shared an Id% higher than the Cut-In value were considered as present and those with Id% lower than the Cut-Off were considered absent. Presence or absence of proteins with shared identity between Cut-Off and Cut-In values was analyzed individually by sinteny. (PDF 407 KB) [file 12864_2013_6171_MOESM1_ESM.pdf]

# eps locus

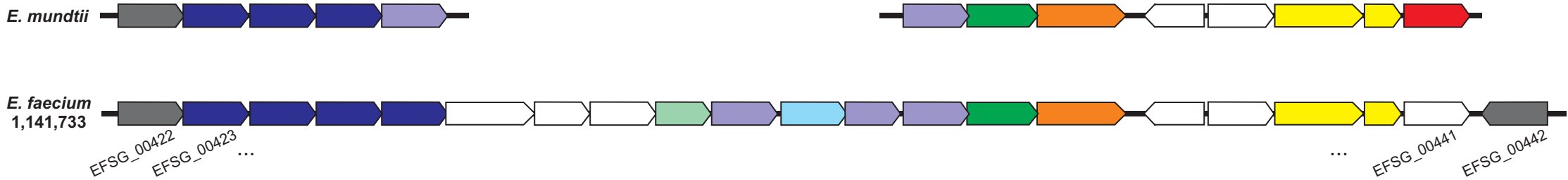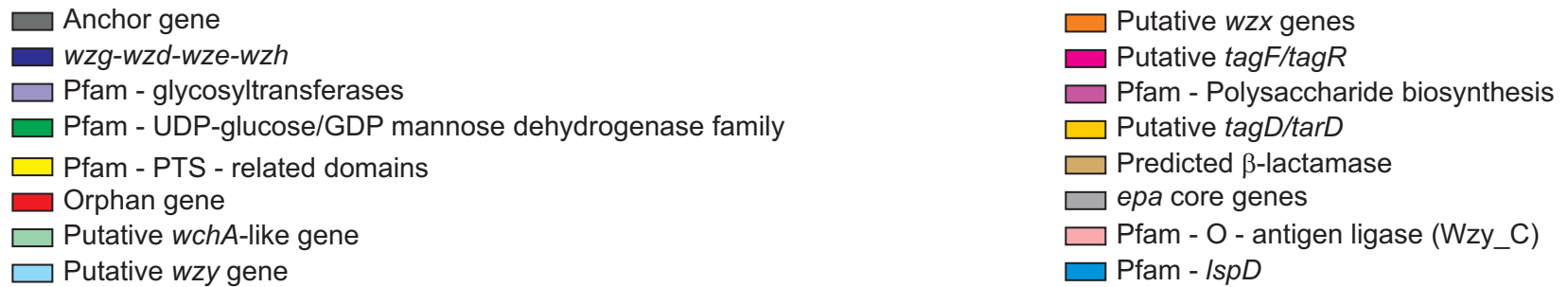

# epa locus

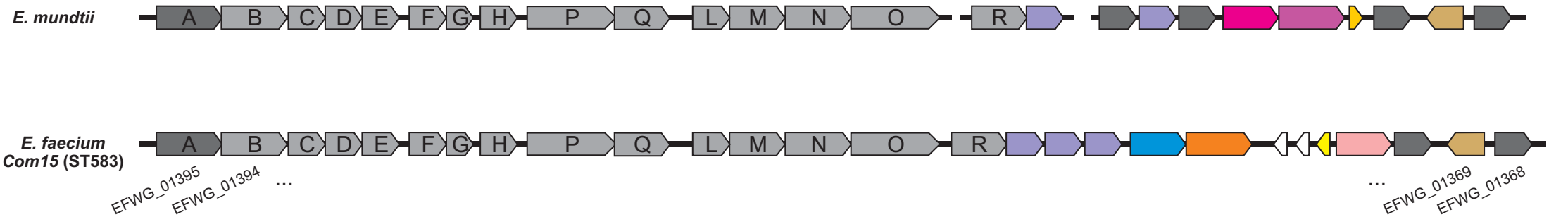

Supplement: Supplementary file 2 — Additional file 2: Table S1: Putative functions associated to horizontal gene transfer. (PDF 405 KB) [file 12864_2013_6171_MOESM2_ESM.pdf]
