# Supplementary material for: Genomic comparative analysis of the environmental Enterococcus mundtii against enterococcal representative species
Source: BMC Genomics. 2014 Jun 18;15(1):489. doi: 10.1186/1471-2164-15-489 (PMC4076982; doi:10.1186/1471-2164-15-489)
Supplement: Supplementary file 5 — Additional file 5: Figure S2: epa and eps gene clusters present in E. mundtti CRL1656. E. faecium 1.141.733 epa genes and E. faecium Com 15 eps genes are shown for comparison. Genes are colored following the code included in the figure. (DOC 59 KB) [file 12864_2013_6171_MOESM5_ESM.doc]

# *Table S3. Two-component systems present in* E. mundtii

| **Group^a^** | **HK/RR** | **HK alignment^b^** | **RR family** | **Homologues in *E. faecalis* V583^c^** | **Role in *E. faecalis*** | **References** |
| --- | --- | --- | --- | --- | --- | --- |
|  |  |  |  |  |  |  |
| **I** | Ia | LQAQINP**H**FLYNTLEYIRM | AraC | EF2219-EF2218 (YesMN) | Antibiotic resistance | Hancock 04 |
|  | Ib | MQAQIQP**H**FIYNTLTSLKF | AraC | EF2219-EF2218 (YesMN) | Antibiotic resistance | Hancock 04 |
|  | Ic | LQAQIIP**H**FIYNSLDAILV | AraC | EF2219-EF2218 (YesMN) | Antibiotic resistance | Hancock 04 |
| **II** | IIa | HRLAREL**H**DSVSQQLFAAM | NarL | EF2912-EF2911 (VraSR) | Antibiotic resistance | Hancock 04 |
|  | IIb | QRIIHEI**H**DHLGHDLTSCL | LuxR | nd-EF2911 (VraR ) |  |  |
|  |  |  |  |  |  |  |
| **IIIa** | IIIa-a | DFVGNVS**H**ELKTPVTSLIG | OmpR | EF1704-EF1703 (PhoRP) | Heat stress resistance | Le Breton 03 |
|  | IIIa-b | ELITNVS**H**DIRTPLTSIIG | OmpR | EF3290-EF3289 (CroSR) | Antibiotic resistance | Hancock 04 |
|  | IIIa-c | QFMADAS**H**EMRTPLTTING | OmpR | EF1261-EF1260 (YclKJ) | Heat and SDS stress resistance | Hancock 04 |
|  | IIIa-d | EFVSNVS**H**ELRTPLTSMRS | OmpR | EF1194-EF1193 (VicKR) | Unknown but essential | Hancock 04 |
|  | IIIa-e | DYIDSWV**H**EIKVPVAASEL | OmpR | EF0927-EF0926 |  |  |
|  | IIIa-f | QFVEDVS**H**ELRTPVAIIEG | OmpR | EF1051 (EtaSR) | NaCl and pH stress resistance/Virulence | Hancock 02 |
|  | IIIa-g | NLLRAIS**H**DLRTPLTAISG | OmpR | EF0570-EF0571 (KdpDE) |  |  |
|  | IIIa-h | EFMQIAS**H**ELKTPVASLMG | OmpR | EF2298-EF2299 (VanSR) |  |  |
|  |  |  |  | EF1863-EF1864 (VncSR) | Heat stress resistance | Le Breton 03 |
|  | IIIa-i | EFVENAS**H**ELRTPLTIIQS | OmpR | nd-EF2299 (VanR) |  |  |
|  | IIIa-j | DFVSNVS**H**EFKTPLATIQG | OmpR | nd-EF1193 (VicR) |  |  |
|  |  |  |  |  |  |  |
| **Other** | Oth-a | QELREFK**H**DYQNLLFSLNS | LytTR | EF1820-EF1822 (FsrCA) | Virulence and biofilm | Hancock 04 |
|  | Oth-b | LEILKFK**H**DYKNILISLEE | LytTR | EF1335-EF1336 |  |  |

# a Grouping is based on the homology of the kinase region surrounding the phosphorylatable histidine.

b Shown is the region surrounding the phosphorylatable histidine (boldface)

c. Listed are the locus numbers, in the order HK-RR, according to the RefSeq database as of June 2013. Shown in parentheses are the proteins names. nd: no homologues detected in *E. faecalis* V583.
